# The effect of a goal-setting strategy with integrated feedback on goal attainment in inflammatory arthritis patients: a mixed method study

**DOI:** 10.1007/s00296-023-05394-3

**Published:** 2023-07-22

**Authors:** K. van Slingerland, P. H. P. de Jong, R. J. E. M. Dolhain, A. Pasma

**Affiliations:** 1 Department of Rheumatology, Erasmus MC, Rotterdam, The Netherlands; 2 Department of Quality and Patient Care, Erasmus MC, Rotterdam, The Netherlands

**Keywords:** Person-centered care, Patient goal setting, Self-management

## Abstract

Patients with Inflammatory Arthritis (IA) often experience difficulties in daily life as a result of their disease. Unfortunately, outpatient consultations in daily practice tend to focus on medical topics, thereby ignoring the impact of the disease on patients’ daily lives. Patient-Reported Outcomes (PROs) can be used to understand this impact, but they are not enough for offering person-centered care. Because the patient’s true values and goals can only be ascertained during a proper conversation, which should include both medical as well as patient goals. Therefore, the aim of the study is to evaluate the effect of a goal management strategy with integrated feedback on goal attainment and Health-Related Quality of Life(HRQoL) in IA patients. IA patients with an active disease were given the opportunity to set and follow-up goals. In addition to goal setting, patients were asked to complete online questionnaires on various PROs, including HRQoL. Ninety-two IA patients participated in the study. The mean age was 51 years and most of them had rheumatoid arthritis. A total of 302 patient goals were set, of which 32% were achieved. In the entire population, HRQoL did not change over time, but patients who did not meet their goals tended to report a lower HRQoL. Incorporating a feedback mechanism in a goal-setting strategy has a positive effect on goal attainment. Yet no effect was seen on HRQoL, but this may due to the fact that general HRQoL measurement are not sensitive or specific enough to detect changes that are accompanied with goal setting and attainment.

## Background

Suffering from a chronic condition, such as inflammatory arthritis (IA), can have a major impact on daily life. It can lead to limitations in various areas of life due to pain and treatment of the rheumatic disease. As a result, patients experience a constant struggle to self-manage the disease and cope with life [1]. To aid patients in self-management, healthcare is increasingly shifting towards a person-centered care (PCC) approach, which is specifically recommended for patients with chronic conditions [2]. For healthcare professionals, knowledge on how to facilitate PCC during consultation is very important to address the problems patients experience. Currently, this knowledge is lacking, but at the same time, we do not know the added value of PCC on patients’ lives.

PCC is care that focuses on the needs of the individual patient [3–5]. Within PCC people’s preferences, needs and values should guide clinical decisions, and the given care should be respectful, responsive to the individual patient and include a holistic focus [5, 6]. PCC should support patients’ realistic health and life goals and self-management [3, 5]. In daily practice, however, outpatient clinic consultations focus on medical topics rather than applying a holistic care approach, and thus ignoring the disease impact on daily life [7, 8]. Focusing on the problems patients experience in daily life and setting goals to overcome them are very important in PCC [9]. Setting and achieving goals can create a positive feeling and increase the chance of treatment success [10]. Therefore, for optimal treatment success, physicians and patients should define treatment goals together, in which particular attention should be payed to the patient’s life context and priorities [11, 12].

To understand the impact of IA on patients' lives, patient reported outcomes (PROs) can be used [13]. A PRO is a representation of a patients’ health status that comes directly from the patient [14]. The use of PROs in consultations is feasible, because it gives healthcare providers insight into the disease impact and it may facilitate shared decision-making (SDM). Moreover, it is associated with high patient satisfaction and treatment confidence [13]. However, it is not enough to use PROs and discuss them during the consultation, as this cover not all aspects of PCC. Patients’ true values and goals can only be found out during a good conversation between the patient and the healthcare provider and cannot be replaced by a questionnaire [15].

It is assumed that providing PCC through goal setting improves patients' quality of life. In addition goal attainment may be improved by incorporating feedback strategies. Unfortunately, goal setting usually do not include techniques for feedback and monitoring. It is also unknown in which domains goals are set. In line with this, there are no studies within rheumatology—to our knowledge—on encouraging patients to set goals beyond the medical scope.

### Objective

Therefore, the aim of this study is to evaluate the effect of a goal management strategy with integrated feedback on goal attainment and Health-Related Quality of Life (HRQoL) in Inflammatory Arthritis (IA) patients. In addition, we explored what type of goals are set. We hypothesize that by encouraging IA patients to set goals through a personal interview and an automatic feedback reminder, more goals are attained and HRQoL improves.

## Methods

### Study design

A mixed method evaluation study was conducted in IA patients with active disease to examine the effect of a goal management strategy with integrated feedback on goal attainment and HRQoL. The mixed method is reflected in the types of questions asked and analyses performed. Quantitative analyses were performed on data obtained from the Immune Mediate Inflammatory Disease (IMID) registry, a prospective cohort study in which IMID patients are followed with online PRO questionnaires. The qualitative method is reflected in the open-ended questions about patient goals and experiences with the goal-setting process, and the qualitative analyses conducted. The study was performed at the outpatient clinic of the department of rheumatology of the Erasmus MC, an academic hospital in Rotterdam, The Netherlands. The study was approved by the Medical Ethics Committee of the Erasmus MC. They considered the study not subject to Dutch law and provided a waiver (MEC-2019-0643).

### Study participants

Patients who visited the rheumatologist or nurse practitioner (NP) from July 2020 to July 2021 and were willing to set goals, were invited to participate in this study. In addition, patients were asked to enroll in the IMID registry. Inclusion criteria were: (1) age ≥ 18 years, (2) diagnosed with an inflammatory arthritis (i.e. Rheumatoid arthritis, psoriatic arthritis or Spondyloarthritis), (3) having active disease which was already treated with a DMARD and (4) being able to understand, speak and write in Dutch.

The study was powered to detect a 0.08 increase in HRQoL, measured with the 5-level EuroQol (EQ-5D-5L). Following assumptions were made: (1) mean EQ-5D-5L at baseline was 0.60 with a standard deviation of 0.25, which was based upon data from the tREACH trial. If using a significance level of α = 0.05 and a power of 80%, 90 IA are needed allowing for 20% dropout.

### Study procedures

**Fig. 1:**
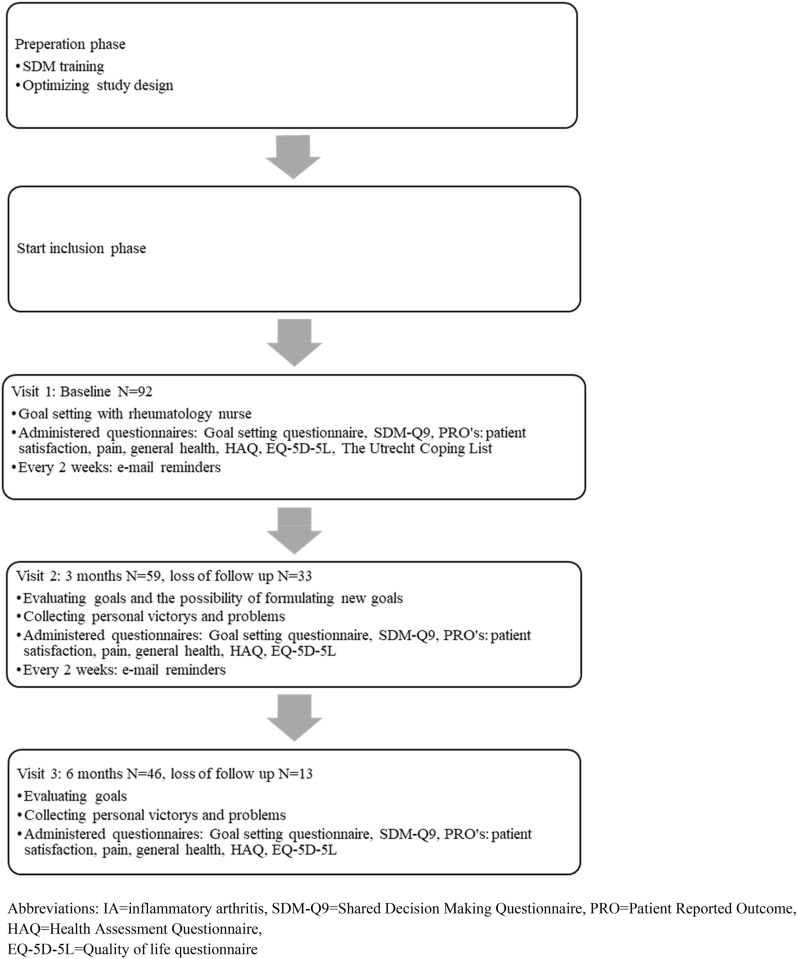
Study design

In preparation for the study, the involved rheumatologists and rheumatology nurses (RNs) completed a training on communication techniques which are needed for goal setting and SDM. The study design and logistics were presented to the patient panel and their feedback was used to optimize the study design (Fig. 1).

After informed consent, IA patients were asked to formulate a maximum of three achievable goals, with assistance of the RN. Patients worked independently on the goals for 6 months. Patients were motivated to work on their goals by an automatically generated e-mail reminder which they received every two weeks. After 3 and 6 months, the RN discussed with the patient whether the goals had been achieved or not and what problems they might have experienced. If goals were achieved after 3 months, new goals could be formulated for the next 3 months. At each visit (baseline, 3 and 6 months), patients received an e-mail with a link to the online questionnaires.

### Data collection

Demographic data and disease characteristics such as diagnose, disease duration, erosion and biological use were collected from patient records.

Formulated goals were registered in the electronic clinical report form. Patients’ goals were classified into the 14 domains of the Self-management Web, extended with the domains pain and energy [16]. The perceived victories and personal difficulties during the goal-setting process were classified into larger subcategories. The formulated goals, victories and difficulties were independently categorized by one of the participating RN and the investigator. Discordant cases were discussed and re-categorized after consensus was reached (Qualitative measurements).

At baseline, and after 3 and 6 months online questionnaires on goal setting, PROs and SDM were taken (Quantitative measurements). PRO questionnaires included health related quality of life (HRQoL), general health, pain, patient satisfaction and activity limitations. In addition, a questionnaire on coping styles was taken at baseline.

The goal-setting questionnaire contains 10 statements about social support, caregiver support, intrinsic motivation, internal locus of control and perceived self-efficacy in achieving the goal. The items can be scored on a four-point Likert scale, from totally disagree (1) to totally agree (4). A score ≥ 3 implied that the patient agreed with the statement. After 3 and 6 months, patients also completed questionnaires on the extent to which they had achieved their goals. This could be scored using a five-point Likert scale from totally not achieved (1) to absolutely achieved (5). A score ≥ 4 was defined as achieved. Patients were also asked to describe what their greatest personal victory was after 3 and 6 months and what problems they experienced during that period.

The five-level EuroQol (EQ-5D-5L) is an instrument that scores quality of life on 5 health dimensions: mobility, self-care, daily activities, pain/discomfort and anxiety/depression. The calculated score reflects the perceived quality of life [17].

General health (GH), pain and patient satisfaction were measured with a visual analogue score (VAS), ranging from 0 to 100. For GH and pain, a lower score indicates less pain and a better health status, while a higher score for patient satisfaction indicates greater satisfaction with treatment.

Activity limitations is measured with the validated Dutch version of the Health Assessment Questionnaire (HAQ) [18]. The questionnaire includes 20 specific functions grouped into 8 categories, namely dressing and grooming, arising, eating, walking, personal hygiene, reaching, gripping, and other activities. The statements can be scored by a four-point Likert scale, from no difficulty (0) to unable (3). The mean of these scores forms a physical functioning score ranging from 0 to 3 and a higher score corresponds with more functional impairment.

The Utrecht Coping List (UCL) is used to measure average coping behavior and consists of 47 items divided into 7 subscales, namely active approach, palliative response, avoidance, seeking social support, passive response pattern, expression of emotions and comforting thoughts. Each item can be scored by a four-point Likert scale. A higher score on any of the subscales indicates more frequent use of this coping style [19].

The patients’ perceived level of SDM was measured with the SDM-Q9. The SDM-Q9 consists of nine items, which can be scored using a six-point Likert scale. Higher SDM-Q9 scores indicate a higher level of perceived SDM [20].

### Data analysis

The goal-setting process was evaluated with descriptive statistics. It was evaluated how many goals were achieved, how the degree of SDM developed. The types of goals and the victories and personal difficulties patients experienced during the process were qualitatively analyzed.

Several additional analyses were conducted. First, we examined whether PROs improved or worsened during the goal-setting process. Subsequently, we evaluated whether there was a trend between the PROs (over time) of IA patients who achieved their goals and those who did not. Finally, we explored whether different coping styles affected goal attainment. Means are presented for normally distributed data and medians for non-normally distributed data. Statistical analyses were performed using Stata version 17.

## Results

### Baseline outcomes

A total of 92 IA patients were included (Table 1). The mean age was 51 years, and the majority was female(60%). Most prevalent diagnosis was rheumatoid arthritis (54%) and 30% of the RA patients included had an erosive disease. The mean disease duration was 5.2 years. A biological was used by 66% of patients.

**Table 1:** Baseline characteristics

Baseline characteristics	Total (*n* = 92)
Gender: Female, *n*(*%*)	55 (60)
Age, *mean*(*sd*)	51 (15)
*Disease characteristics*:	
Diagnosis, *n(%)*	
Rheumatoid arthritis	50 (54)
Erosive disease in RA, *n*(%)	15 (30)
Spondylarthritis	18 (20)
Psoriatic arthritis	19 (21)
Juvenile idiopathic arthritis	3 (3)
Systemic Lupus Erythematosus	2 (2)
Disease duration (years), *mean*(*sd*)	5.2 (7)
Biological use, *n*(*%*)	60 (66)

### Goal setting process

A total of 312 goals were formulated at baseline and after 3 months (Table 2a). Most goals were formulated in the domains lifestyle (14%), pain (13%) and symptoms and side-effects (13%). Less goals were formulated in the domains intimate relationships and sexuality (0.6%) and finances (0.3%). No goals were formulated in the domains family, friends and social network and household chores. Of the 312 goals set, 100 were achieved (32%). Most goals were achieved in the domains emotions and giving meaning to life (60%), daily activities and work (53%) and lifestyle (46%).

**Table 2:** Patient goals, victories and problems

Domains patient goals T0 + T3	*N* (%)	% achieved
**a**. **Patient goals**		
Lifestyle	45 (14)	46
Pain	39 (13)	43
Symptoms and side-effects	39 (13)	27
Leisure activities	37 (12)	27
Dealing with treatment recommendations	31 (10)	25
Energy	23 (7)	30
Daily activities and work	20 (6)	53
Transport and mobility	19 (6)	39
Emotions and giving meaning to life	18 (6)	60
Shared decision-making	15 (5)	42
Illness-related knowledge	13 (4)	40
Self-care	10 (3)	13
Intimate relationships and sexuality	2 (0.6)	0
Finances	1 (0.3)	0
Family, friends and social network	–	
Household chores	–	
Total goals	312 (100)	
Achieved goals	100 (32)	

Domains	Personal victory %	Experienced problems %
**b. Victories and problems**		
Physical	48	45
Mental	14	16
Energy	2	7
Medication	16	11
Daily functioning	14	6
Balance	3	9
Health-related topics	2	2
Life style	2	
Weather		2
Relationship		1

During the goal-setting process, patients encountered both personal victories as well as problems (Table 2b). The victories they experienced were mostly physical (48%) and medication-related (16%). Most problems were experienced in the physical (45%) and mental domain (16%). Examples of goals, personal victories and problems are shown in Table 3.

**Table 3:** Examples of goals, personal victories and experienced problems

**Examples of goals**
*“It is very important for me to be able to keep working, I am self-employed and not working directly affects our income” (male, RA, 38 years old)*
*“I have lost a lot of condition and strength. I would like to have more condition and less pain so that I can enjoy a walk again and look around at the beauty of nature. I’ll start with a small walk, but I’d like to see if bigger trips are possible again”* (male, spondylarthritis, 51 years old)
*“ I would like to lose weight by eating healthier” (female, psoriatic arthritis, 42 years old)*
**Examples of personal victories**
*“I went for a short bike ride again for which I did not have the energy before”* (female, psoriatic arthritis, 25 years old)
*“I am expanding my working hours. At first I came to the office for coffee once a week and now I am able to work 10 h a week. I'm already halfway and it feels so good”* (female, psoriatic arthritis, 45 years old)
**Examples of experienced personal problems**
*“Acceptance of being chronically ill in general”* (male, psoriatic arthritis, 29 years old)
*“My fatigue has to much influence”* (male, psoriatic arthritis, 26 years old)
*“Communication with my treating physician is cumbersome”* (female, RA, 26 years old)

### PRO’s

No difference was seen in health related quality of life, measured with EQ-5D-5L, at baseline 0.63 (sd 0.26) and after 6 months 0.65 (sd 0.23). All other measured PROs also did not differ (Table 4). For example, the VAS GH at baseline was 41 (sd 22), while after 6 months, it was 46 (sd 25). VAS pain was also similar over time, 45 (sd26) at baseline and 44 (sd 24) after 6 months. Patient satisfaction was 66 (sd 21) at baseline versus 67 (sd 22) after 6 months. Finally, the HAQ was 0.90 (sd 0.62) at baseline, while it was 0.92 (sd 0.56) after 6 months.

**Table 4:** PRO’s at baseline and after 6 months, also stratified for IA patients achieving and not achieving their goals

PRO’s:	Baseline *N* = 65	6 months *N* = 52	IA patients with goal attainment 6 months *N* = 47	IA patients without goal attainment 6 months *N* = 5
EQ5D, *mean utility index (sd)*	0.63 (0.26)	0.65 (0.23)	0.67 (0.21)	0.46 (0.34)
VAS Patient satisfaction, *mean(sd)*	66 (21)	67 (22)	69 (21)	52 (31)
VAS Pain, *mean(sd)*	45 (26)	44 (24)	45 (25)	40 (24)
VAS General health, *mean(sd)*	41 (22)	46 (25)	47 (25)	40 (30)
HAQ, *mean(sd)*	0.90 (0.62)	0.92 (0.56)	0.85 (0.51)	1.43 (0.76)

IA patients who did not achieve their goals tend to score worse on HRQoL, patient satisfaction and physical functioning after 6 months (Table 4). In contrast, VAS general health and pain showed no relation with goal attainment.

### Coping styles

When setting goals, there was much perceived support from the treating physician (Table 5). Almost all patients were motivated to achieve their goals and the majority made a plan for it. If patients did not achieve their goals, they knew where to turn to for help. On the other hand, if the goal was achieved, they felt that it was due to their own effort. The patients’ most common coping styles were active approach and comforting thoughts.

**Table 5:** Coping styles

Goal setting questionnaire	
Statements about support from caregiver	% of patients agreed with the statement
My doctor discussed my goal with me	94
My doctor has motivated me to meet my goal	89
*Statements social support*	
If I don’t succeed in working on this goal, I know who to turn to for help	87
I need help to achieve this goal	58
*Statements internal motivation*	
I would like to achieve this goal very much	99
*Statements patient perceived self-efficacy*	
I think I can easily meet this goal	75
I have come up with a plan to meet this goal	81
I don’t really know how to meet this goal	28
*Statements Internal locus of control*	
If I reach my goal I own it mostly to myself	81
If I don’t reach my goal, it’s my own fault	20
*Most common coping styles*:	
Active approach; subscale range 4–28, *mean*(*sd*)	19 (0.4)
Comforting thoughts; subscale range 4–20, *mean*(*sd*)	13 (0.3)

The patient’s perceived degree of SDM at baseline is reflected by a mean SDM-Q9 score of 35.3 (range 0–45). After 6 months, the mean score decreased to 28.3.

## Discussion

This study showed that 32% of IA patients achieved their goals with our goal-setting strategy with integrated feedback. On the other hand, we saw no improvement on the measured generic PROs, such as HRQoL, general health, pain, patient satisfaction and HAQ after 6 months.

To our knowledge, there is no literature on the effect of a goal-setting strategy with integrated feedback on achieving personal goals within rheumatology. However, we did find literature on goal achievement in other disciplines. This particularly concerns the field of physical therapy and rehabilitation. The emphasis in these studies is not on goal attainment, but on the progress that is made within the set goal. [21, 22]. In rehabilitation medicine, the goal attainment scale (GAS) is widely used, which was originally developed for use in mental health care. Within the GAS, for each specific goal a scale is used to formulate a range of likely outcomes, ranging from least to most favorable. These “outcome scales” are formulated in consultation with the patient and are, thus, specific for that individual patient [22, 23]. Although we found no literature on the effect of an integrated feedback mechanism on goal attainment, we do believe it has a positive effect on goal attainment, because feedback and monitoring are essential elements of self-regulation. Without feedback and monitoring, comparisons cannot be made and evaluation of the effects of one's self-regulatory actions is not possible [24, 25].

The formulated patient goals were heterogeneous and covered different facets of daily life [11, 12, 21, 26]. However, most of the formulated goals were mainly symptom-oriented, disease-specific and functional [27]. This is consistent with previous literature showing that the problems IA patients experience in daily life are related to their own disease, such as pain and fatigue [8]. It could be argued that goals in other domains would have been set if RN had been trained to use a supportive tool, such as the Self-Management Web or The Self-Management Identification Tool, to identify which domains patients experience problems on in daily life. Subsequently, the RN could help the patient with setting achievable goals in that specific domain on which the patient could work on independently thereafter [3, 16].

Our study also showed that our goal-setting strategy had no effect on the measured generic PROs. Despite small non-significance differences in HRQoL, patient satisfaction and functional ability in patients who met and did not met their goals, no significant differences were seen in the group as a whole. Our chosen PRO’s might not be appropriate for measuring the effect of a goal-setting strategy, because of their generic nature and that they only measure the impact of the disease. Setting goals might improve self-management and self-efficacy, because patients actively address their problems in daily life and goal attainment might help them overcome these problems as well as help them with managing the disease. Thus, to assess the effectiveness of a goal-setting intervention, outcomes should focus more on measuring patients’ self-management or self-efficacy, or by qualitatively studying the effect of goal attainment on patients’ lives.

This study had some limitations. First, despite the fact that both rheumatologist and RN received the SDM training, only RN guided the patients’ goal setting. Patient goals were not discussed during rheumatologist’s outpatient visits, which may have caused the worsening of the patient’s perceived level of SDM. Secondly, the study was conducted largely in patients with established RA. Outcomes in newly diagnosed IA patients may differ because these patients have more difficulty with disease self-management and to cope with life, as they have less experience with disease management and treatment. Besides early versus established disease, IA diagnosis, for example rheumatoid arthritis versus spondyloarthritis, might also influence the results, which we do not except. However, due to the small numbers, we could validate our assumption. Finally, most of the patients who were included were eager to participate and, thus, motivated to achieve their goals, which might have caused a selection bias. However, despite their eagerness, the dropout during follow-up was large.

In conclusion, incorporating a feedback mechanism in a goal-setting strategy seems to have a positive effect on goal attainment. On the other hand, no effect was seen on HRQoL and other generic PROs and, therefore, we recommend specific outcomes, or qualitative assessment of outcomes, in goal-setting studies.

## Conclusion

Our study shows that a goal-setting strategy with integrated feedback and guidance of rheumatology nurses can support the delivery of person-centered care as well as goal attainment. However, the process is time-consuming and health professionals should be trained in goal setting by using, for example, the Self-Management web. The Self-Management Web is an online support tool that helps patients organize and prioritize experienced problems in different domains and subsequently helps them formulate their goals, preferably in preparation of the outpatient clinic visit, might circumvent aforementioned problems. Moreover, it might assist in concretizing patients’ goals during the consultation, which could save time and, therefore, lower the barrier for implementation.

## Data Availability

Data available on request from the authors.
